# Sacubitril valsartan combined with bisoprolol reduces doxorubicin-induced cardiotoxicity in rats by attenuating oxidative stress

**DOI:** 10.3389/ebm.2026.10946

**Published:** 2026-04-28

**Authors:** Ping Liu, Hui Yang, Runqi Li, Hui Huang, Min Xu

**Affiliations:** 1 Laboratory Department, Xinhua Hospital of Ili Kazak Autonomous Prefecture, Yining, Xinjiang, China; 2 Department of Cardiology, The First Affiliated Hospital of Shihezi University, Shihezi, Xinjiang, China; 3 Department of Nephrology, The First Affiliated Hospital of Shihezi University, Shihezi, Xinjiang, China; 4 Department of Cardiology, Xinhua Hospital of Ili Kazak Autonomous Prefecture, Yining, Xinjiang, China; 5 Department of Critical Care Medicine, Xinhua Hospital of Ili Kazak Autonomous Prefecture, Yining, Xinjiang, China

**Keywords:** adriamycin, bisoprolol, cardiotoxicity, oxidative stress, sacubitril valsartan

## Abstract

Doxorubicin-induced cardiotoxicity remains a leading cause of mortality among cancer patients, with oxidative stress serving as a central pathogenic mechanism. This study investigated whether combination therapy with sacubitril valsartan and bisoprolol attenuates doxorubicin-induced cardiotoxicity through modulation of oxidative stress pathways. Sixty male Sprague-Dawley rats were randomized into five groups: control, doxorubicin (DOX), bisoprolol (1.0 mg/kg/d), sacubitril valsartan (30 mg/kg/d), and combination therapy. All groups except control received intraperitoneal DOX (2.5 mg/kg weekly for 5 weeks). Cardiac function was assessed by echocardiography, myocardial injury by histopathology and enzyme levels (CK-MB, cTnI, BNP), and oxidative stress by ROS fluorescence, MDA, and SOD. Protein expression of Nrf2, HO-1, and Keap1 was analyzed by Western blot. DOX administration significantly impaired cardiac function, induced myocardial structural damage, elevated cardiac enzymes and oxidative stress markers, and downregulated Nrf2 pathway proteins compared to controls (all P < 0.05). All treatment groups significantly attenuated these abnormalities versus DOX (all P < 0.05), with combination therapy demonstrating superior cardioprotection evidenced by greatest improvement in LVEF (68.74 ± 6.87% vs. 50.26 ± 6.11%, P < 0.05), lowest cardiac enzyme levels, and most robust restoration of Nrf2 pathway expression. These findings demonstrate that sacubitril valsartan combined with bisoprolol effectively reduces doxorubicin-induced cardiotoxicity in rats by activating Nrf2-mediated antioxidant responses, providing experimental evidence for a potentially synergistic prophylactic strategy.

## Impact statement

Chemotherapy with doxorubicin saves lives, but its heart-damaging side-effects limit how much medicine patients can safely receive. We show that a simple, low-cost pill combining two common heart drugs—sacubitril/valsartan and bisoprolol—prevents this damage in rats by switching on the body’s own antioxidant defense system. This is the first demonstration that the duo works better than either drug alone, restoring heart function and lowering injury markers after just 18 days of treatment. The findings give clinicians an immediately translatable strategy to protect cancer patients’ hearts without altering the tumor-killing power of doxorubicin, potentially allowing higher, more effective doses while reducing heart failure risk.

## Introduction

Anthracyclines are widely used chemotherapeutic agents for the treatment of both solid tumours and haematological malignancies, yet anthracycline-induced cardiotoxicity remains one of the leading causes of mortality among patients with cancer [1–3]. Doxorubicin, one of the most commonly prescribed anthracyclines, has potent antitumour efficacy; however, its clinical use is substantially limited by dose-dependent and largely irreversible cardiotoxic effects. These effects lead to myocardial injury and may ultimately result in arrhythmias, myocardial infarction, and ventricular hypertrophy, thereby markedly increasing long-term cardiovascular mortality [4–6]. Current clinical guidelines recommend the prophylactic use of cardioprotective agents in patients receiving anticancer therapies with established cardiotoxic potential [7, 8]. Nevertheless, effective prevention and treatment strategies for doxorubicin-induced cardiotoxicity remain under active investigation. Previous studies have suggested that angiotensin-converting enzyme inhibitors, statins, and β-adrenergic blockers may confer varying degrees of cardioprotection, although stronger evidence from well-designed studies is still required [9, 10]. Sacubitril/valsartan, an angiotensin receptor–neprilysin inhibitor, has been shown to improve cardiac pump function, and experimental studies indicate that it can attenuate anthracycline-induced cardiotoxicity in rats by modulating oxidative stress–related pathways [11]. Bisoprolol, a selective β-adrenergic blocker widely used in the treatment of chronic heart failure [12], has also demonstrated cardioprotective effects. Ma Yuru et al. [13] reported that bisoprolol alleviates myocardial fibrosis, reduces ventricular hypertrophy, and improves cardiac function in rats with chronic heart failure through inhibition of pathological signalling pathways. Despite these findings, evidence regarding the combined effects of bisoprolol and sacubitril/valsartan on doxorubicin-induced cardiotoxicity is lacking. The mechanisms underlying doxorubicin-associated cardiac injury have not yet been fully elucidated and are believed to involve multiple pathological processes, including oxidative stress, mitochondrial dysfunction, ferroptosis, autophagy, and apoptosis. Among these mechanisms, oxidative stress is widely regarded as a central contributor to doxorubicin-induced cardiotoxicity [14–16]. Against this background, the present study aimed to investigate whether the combination of sacubitril/valsartan and bisoprolol mitigates doxorubicin-induced cardiotoxicity in rats through modulation of oxidative stress pathways. The findings are intended to provide mechanistic insight and experimental evidence to support novel combination strategies for the prevention of anthracycline-related cardiotoxicity.

## Materials and methods

### Experimental animals

A total of 60 male Sprague–Dawley rats were obtained from the Guangdong Institute for Product Quality Supervision and Inspection (license no. SYXK [Yue] 2023-0181). The animals were 6–8 weeks old and weighed 180–220 g; they were housed in groups at the institutional animal experimental centre under controlled conditions (temperature 23–27 °C, relative humidity 50%–60%, and a 12-h light/12-h dark cycle). All rats were allowed a one-week acclimatisation period before the start of the experiment. Throughout the study, animals had free access to standard chow and water.

### Reagents and instruments

Doxorubicin was purchased from Zhejiang Hisun Pharmaceutical Co., Ltd.; bisoprolol from Hangzhou Minsheng Pharmaceutical Co., Ltd.; and sacubitril/valsartan from Novartis (China) Biomedical Research Co., Ltd. Haematoxylin–eosin staining reagents were obtained from Beyotime Biotechnology (Shanghai, China). Commercial ELISA kits for CK-MB, SOD, BNP, cTnI, and MDA were purchased from Shanghai Enzyme-linked Biotechnology Co., Ltd. The catalog numbers and specifications were as follows: CK-MB ELISA kit (Cat# ML037723), SOD ELISA kit (Cat# ML077379), BNP ELISA kit (Cat# ML059422), cTnI ELISA kit (Cat# 059111), MDA ELISA kit (Cat# ML077384). Primary antibodies against nuclear factor erythroid 2-related factor 2 (Nrf2), haem oxygenase-1 (HO-1), Kelch-like ECH-associated protein 1 (Keap1), and GAPDH were obtained from Abcam (Cambridge, UK). The following antibodies were used: anti-Nrf2 (ab137550, rabbit monoclonal, 1:1000, validated for rat), anti-HO-1 (ab68477, rabbit monoclonal, 1:1000, validated for rat), anti-Keap1 (ab227828, rabbit monoclonal, 1:1000, validated for rat), and anti-GAPDH (ab181602, rabbit monoclonal, 1:5000, validated for rat). HRP-conjugated secondary antibody (ab205718, goat anti-rabbit IgG, 1:5000) was also purchased from Abcam.

Cardiac function was assessed using a VisualSonics Vevo 2100 high-resolution small-animal ultrasound system (FUJIFILM VisualSonics, Canada). Flow cytometric analyses were performed with an Attune™ NxT flow cytometer (Thermo Fisher Scientific, USA).

### Experimental grouping and model establishment

Sixty rats were randomly assigned to five groups (n = 12 per group): normal control (Control), doxorubicin (DOX), bisoprolol (Bisoprolol), sacubitril/valsartan (Valsartan), and combination therapy (Combine). Rats in the Bisoprolol, Valsartan, and combination groups received daily oral gavage of the respective drugs for five consecutive weeks, starting 1 week prior to the first doxorubicin administration and continuing throughout the entire experimental period. Specifically, the Bisoprolol group received bisoprolol (1.0 mg/kg), the Valsartan group received sacubitril/valsartan (30 mg/kg), and the combination group received both sacubitril/valsartan (30 mg/kg) and bisoprolol (1.0 mg/kg) once daily by oral gavage. Rats in the Control and DOX groups received an equivalent volume of 0.9% normal saline by oral gavage by oral gavage daily during the same period.

This pretreatment period was designed to ensure that steady-state plasma concentrations of the cardioprotective agents were achieved before the first doxorubicin dose, consistent with standard prophylactic protocols for preventing chemotherapy-induced cardiotoxicity.

One hour after gavage, rats in all groups except the Control group were administered doxorubicin intraperitoneally at a dose of 2.5 mg/kg once weekly for 5 weeks (cumulative dose 15 mg/kg). Rats in the Control group received an equivalent volume of 0.9% normal saline by intraperitoneal injection, and general health status was monitored throughout the experimental period.

### Transthoracic echocardiography

Twenty-four hours after the final gavage, rats were fasted for 6 h before echocardiographic assessment. Anaesthesia was induced by intraperitoneal injection of 2% sodium pentobarbital (3 mL/kg), after which the chest hair was removed and the animals were placed supine with limbs secured to the examination platform to optimise intercostal access. Cardiac function was evaluated using a high-resolution small-animal echocardiography system.

Two-dimensional guided M-mode images were obtained from the parasternal long-axis view at the level of the papillary muscles. Left ventricular end-systolic diameter (LVESD) and left ventricular end-diastolic diameter (LVEDD) were measured according to the American Society of Echocardiography guidelines. Left ventricular ejection fraction (LVEF) was calculated using the Teichholz formula: LVEF (%) = [(LVEDD^3^ - LVESD^3^)/LVEDD^3^] × 100. Left ventricular fractional shortening (LVFS) was calculated as: LVFS (%) = [(LVEDD - LVESD)/LVEDD] × 100. All measurements were averaged over three consecutive cardiac cycles.

### Haematoxylin–eosin (H&E) staining

Following echocardiographic examination, rats were euthanised by exsanguination under deep anaesthesia induced by an intraperitoneal injection of 2% sodium pentobarbital (100 mg/kg body weight). Following the confirmation of deep anesthesia (absence of pedal reflexes), the thoracic cavity was opened, blood samples were collected from the inferior vena cava, and the heart was rapidly excised to isolate myocardial tissue. Approximately 50 mg of left ventricular myocardium from each rat was fixed in paraformaldehyde, embedded, and sectioned at a thickness of 4 μm. For each animal, three non-consecutive sections were collected and stained with hematoxylin and eosin (H&E) for histopathological evaluation.

After deparaffinisation in xylene and rehydration through graded ethanol, sections were stained with haematoxylin for 10 min, differentiated briefly in acid alcohol, blued, and counterstained with eosin for 2 min. After dehydration and mounting with neutral resin, histopathological changes in myocardial tissue were examined under light microscopy.

For semi-quantitative analysis, myocardial injury was scored based on the following criteria modified from previous studies [Reference]: 0 = normal myocardium with regularly arranged cardiomyocytes and no visible damage; 1 = mild focal myofibrillar loss or cytoplasmic vacuolization involving <25% of the field; 2 = moderate multifocal myofibrillar loss, cytoplasmic vacuolization, or interstitial edema involving 25–50% of the field; 3 = severe confluent myofibrillar disorganization, nuclear pyknosis, karyorrhexis, or inflammatory cell infiltration involving >50% of the field. Five randomly selected fields per section (15 fields per animal) were evaluated, and the average score was calculated for each rat.

### Measurement of myocardial enzymes and oxidative stress markers by ELISA

Myocardial tissue (30 mg) and blood samples were collected from rats in each group following the procedures described above. Blood samples were allowed to clot at room temperature for 30 min before centrifugation at 3,000 × g for 10 min (radius 12 cm) to obtain the supernatan. All samples were processed within 2 h of collection to ensure optimal analyte stability. The supernatants from myocardial homogenates and serum samples were used for quantitative determination of CK-MB, MDA, cTnI, BNP and SOD. All measurements were performed using commercially available ELISA kits, strictly according to the manufacturers’ instructions. All ELISA assays were completed within 4 weeks of sample collection, and samples were stored at −80 °C during this period.

### Assessment of reactive oxygen species by flow cytometry

Serum samples: Blood samples were centrifuged at 3,000 × g for 10 min (radius 12 cm) to obtain serum within 1 h of collection. To detect reactive oxygen species (ROS) levels in circulating extracellular vesicles (EVs) enriched fractions, we adapted a previously published method [17]. Briefly, 50–100 μL of serum was incubated with an equal volume of DCFH-DA working solution (diluted in serum-free PBS, final concentration 10 μmol/L) in the dark at 37 °C for 30 min. After incubation, samples were diluted with PBS and immediately analyzed by flow cytometry using an Attune™ NxT flow cytometer (Thermo Fisher Scientific, USA). The EV-enriched particle population was gated based on characteristic forward and side scatter properties, as previously described [17]. It should be noted that this scatter-based gating approach, while widely used for EV-rich fractions, cannot definitively exclude potential contributions from other submicron particles such as lipoproteins or protein aggregates that may share similar scatter characteristics. DCF fluorescence intensity within this gate was quantified to represent serum ROS levels. A total of 10,000 EV events were acquired per sample, and results were expressed as mean fluorescence intensity.

Myocardial tissue samples: Fresh myocardial tissue was enzymatically dissociated using type II collagenase and trypsin, followed by incubation at 37 °C for 30 min with gentle agitation every 10 min. Digestion was terminated by the addition of serum-containing medium, and the cell suspension was filtered through a 40 μm cell strainer to remove undigested tissue fragments, then centrifuged at 3,000 × g for 10 min (radius 12 cm). The total processing time from tissue collection to cell isolation was approximately 45 min. The resulting cell pellet was resuspended in pre-cooled PBS and incubated with DCFH-DA working solution (final concentration 10 μmol/L) at 37 °C for 45 min, with gentle mixing every 10 min. After incubation, cells were washed twice with PBS, resuspended in 500 μL PBS, and intracellular ROS fluorescence was measured by flow cytometry. The entire procedure from tissue collection to flow cytometry analysis was completed within 90 min. A total of 10,000 live cell events were acquired per sample, and results were expressed as mean fluorescence intensity. The analysis was performed on total live cells gated based on forward/side scatter properties, without discrimination of specific cardiac cell subtypes.

### Western blot analysis of Nrf2, HO-1, and Keap1

Approximately 30 mg of myocardial tissue from each rat was lysed in ice-cold lysis buffer for 30 min on ice. Lysates were centrifuged at 12,000 × g for 15 min (radius 12 cm), and the supernatant was collected for protein quantification using the BCA assay within 2 h of extraction. Equal amounts of protein (30 μg per lane) were mixed with loading buffer at a 1:4 ratio, denatured at 95 °C for 5 min, and separated by SDS–PAGE using stacking and resolving gels. Electrophoresis was performed at 80 V until proteins entered the resolving gel and then continued at 120 V until clear separation of the molecular weight marker was achieved. Proteins were transferred onto PVDF membranes, which were blocked with 5% non-fat milk for 60 min at room temperature. Membranes were then incubated overnight at 4 °C with primary antibodies against Nrf2 (1:1,000), HO-1 (1:1,000), Keap1 (1:1,000), and GAPDH (1:5,000), followed by incubation with appropriate secondary antibodies (1:5,000) for 60 min at room temperature. Protein bands were visualised using enhanced chemiluminescence, and band intensities were quantified using ImageJ software.

### Statistical analysis

All data were analysed using SPSS software (version 25.0). Continuous variables that conformed to a normal distribution are presented as mean( $\bar{x}$ ±s). Comparisons among multiple groups were performed using one-way analysis of variance (ANOVA), followed by the least significant difference (LSD) t-test for *post hoc* pairwise comparisons. A two-sided P value <0.05 was considered to indicate statistical significance.

## Results

### General condition of rats in each group

Rats in the Control group exhibited a glossy coat, normal food intake and activity, rapid responses, normal defecation, and good overall vitality. In contrast, rats in the DOX group showed dull fur, markedly reduced food intake and spontaneous activity, sluggish responses, and an overall lethargic appearance. Compared with the DOX group, general condition was noticeably improved in the Bisoprolol, Valsartan, and combination groups. The most pronounced improvement was observed in the combination group.

### Echocardiographic parameters

**FIGURE 1:**
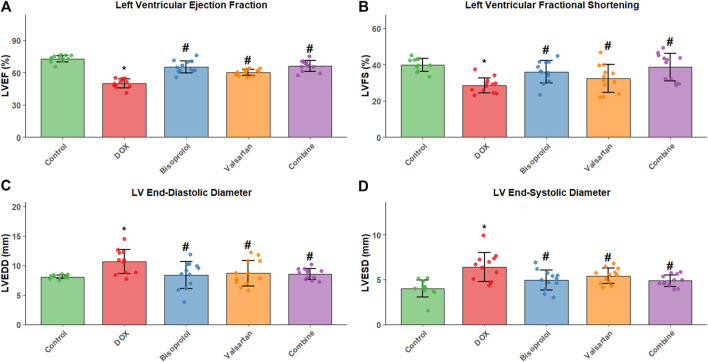
Echocardiographic assessment of left ventricular function in each group. Bar graphs showing **(A)** left ventricular end-diastolic diameter (LVEDD), **(B)** left ventricular end-systolic diameter (LVESD), **(C)** left ventricular ejection fraction (LVEF), and **(D)** left ventricular fractional shortening (LVFS) in Control, DOX, Bisoprolol, Valsartan, and combination groups (n = 12 per group). Data are presented as mean ± SD. *P < 0.05 vs. Control group; #P < 0.05 vs. DOX group. The combination therapy significantly improved cardiac function compared to monotherapy groups.

Compared with the Control group, rats in the DOX group showed significant increases in left ventricular end-diastolic diameter (LVEDD) and left ventricular end-systolic diameter (LVESD), accompanied by marked reductions in left ventricular fractional shortening (LVFS) and left ventricular ejection fraction (LVEF) (all P < 0.05). Relative to the DOX group, LVEDD and LVESD were significantly reduced in the Bisoprolol, Valsartan, and combination groups, whereas LVFS and LVEF were significantly increased (all P < 0.05). Among these treatments, the combination therapy produced the greatest improvement in cardiac function (Figure 1).

### Histopathological changes in myocardial tissue

**FIGURE 2:**
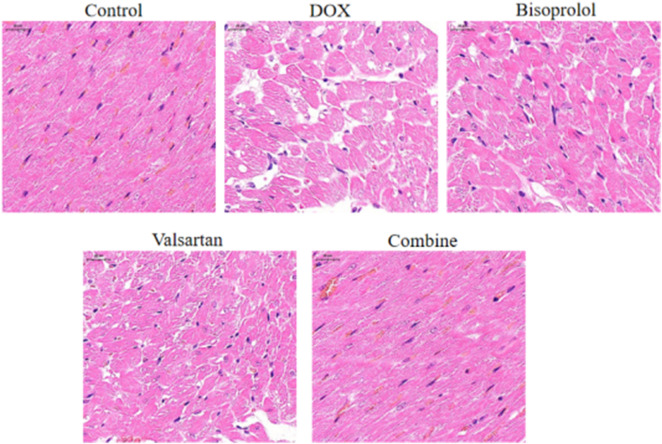
Histopathological assessment of doxorubicin-induced myocardial injury and the protective effects of bisoprolol, sacubitril/valsartan, and their combination. Representative hematoxylin and eosin (H&E) stained sections of left ventricular myocardium from each experimental group (n = 6 per group; 3 sections per animal). The Control group shows normal myocardial architecture with regularly arranged cardiomyocytes, clear striations, and well-defined nuclei and cytoplasm. The DOX group exhibits severe myocardial injury characterized by disorganized myofibrils, extensive cardiomyocyte necrosis, nuclear pyknosis/fragmentation, and prominent inflammatory cell infiltration. The Bisoprolol group shows moderate improvement with reduced myofibrillar disorganization. The Valsartan group demonstrates similar moderate improvement. The combination group shows the most substantial histological improvement with near-normal architecture and minimal residual damage. Scale bar = 50 μm. The lower panel shows semi-quantitative myocardial injury scores. Data are presented as mean ± SD (n = 6 per group). P < 0.05 vs. Control group; #P < 0.05 vs. DOX group; †P < 0.05 vs. Bisoprolol and Valsartan groups (one-way ANOVA followed by LSD t-test).

HE staining revealed normal myocardial architecture in the Control group, with regularly arranged cardiomyocytes, clear striations, and well-defined nuclei and cytoplasm, without evidence of edema or inflammatory infiltration (Figure 2, Control panel). In contrast, the DOX group exhibited severe myocardial injury characterized by disorganized myofibrils, nuclear pyknosis or fragmentation, extensive cardiomyocyte necrosis, marked interstitial edema, and prominent inflammatory cell infiltration (Figure 2, DOX panel).

Myocardial damage was attenuated to varying degrees in the Bisoprolol, Valsartan, and combination groups compared with the DOX group (Figure 2, Bisoprolol, Valsartan, and Combine panels). Semi-quantitative histological scoring confirmed these observations: the DOX group showed significantly higher myocardial injury scores compared with the Control group (2.85 ± 0.42 vs. 0.32 ± 0.18, P < 0.001). All treatment groups significantly reduced injury scores versus the DOX group (Bisoprolol: 1.68 ± 0.35; Valsartan: 1.54 ± 0.31; Combine: 0.86 ± 0.24; all P < 0.05 vs. DOX), with the combination therapy showing the most substantial histological improvement and the lowest injury score among treatment groups (P < 0.05 vs. monotherapy groups). The most substantial histological improvement was observed in the combination group, with near-normal myocardial architecture and minimal residual damage (Figure 2, Combine panel).

### Myocardial enzyme levels in cardiac tissue

**FIGURE 3:**
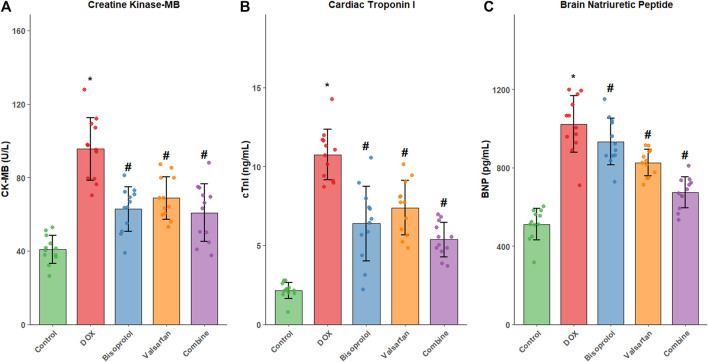
Myocardial enzyme levels in cardiac tissue across experimental groups. **(A)** Levels of creatine kinase-MB (CK-MB), **(B)** cardiac troponin I (cTnI), and **(C)** brain natriuretic peptide (BNP) were measured by ELISA in myocardial homogenates from Control, DOX, Bisoprolol, Valsartan, and combination groups (n = 12 per group). Data are expressed as mean ± SD. *P < 0.05 vs. Control group; #P < 0.05 vs. DOX group. The combination treatment resulted in the most pronounced reduction in cardiac enzyme levels.

Compared with the Control group, levels of creatine kinase-MB (CK-MB), cardiac troponin I (cTnI), and brain natriuretic peptide (BNP) were significantly elevated in the DOX group (all P < 0.05). In contrast, CK-MB, cTnI, and BNP levels were significantly reduced in the Bisoprolol, Valsartan, and combination groups relative to the DOX group (all P < 0.05). Consistent with the functional and histological findings, the greatest reduction in myocardial enzyme levels was observed in the combination group (Figure 3).

### ROS fluorescence intensity in myocardial tissue and serum

**FIGURE 4:**
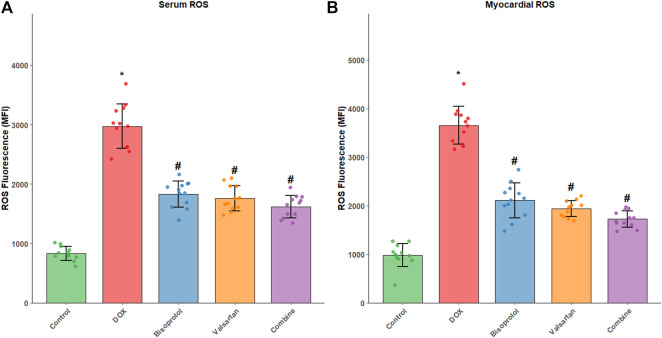
Reactive oxygen species (ROS) levels in serum and myocardial tissue. **(A)** ROS fluorescence intensity in serum-derived extracellular vesicles; **(B)** ROS fluorescence intensity in myocardial cells. ROS fluorescence intensity was measured by flow cytometry in serum-derived extracellular vesicles and myocardial cells from each group (n = 12 per group). Data are shown as mean fluorescence intensity ± SD. *P < 0.05 vs. Control group; #P < 0.05 vs. DOX group. The combination therapy exhibited the greatest reduction in ROS levels in both compartments.

Compared with the Control group, rats in the DOX group exhibited significantly increased reactive oxygen species (ROS) fluorescence intensity in both myocardial tissue and the EV-enriched serum fraction (P < 0.05). In contrast, ROS fluorescence intensity was markedly reduced in the Bisoprolol, Valsartan, and combination groups compared with the DOX group (all P < 0.05), with the combination therapy showing the most pronounced reduction (Figure 4). Myocardial ROS levels represent the average fluorescence of total live cells, as specific cell-type markers were not used in this study.

### Oxidative stress markers in myocardial tissue and serum

**FIGURE 5:**
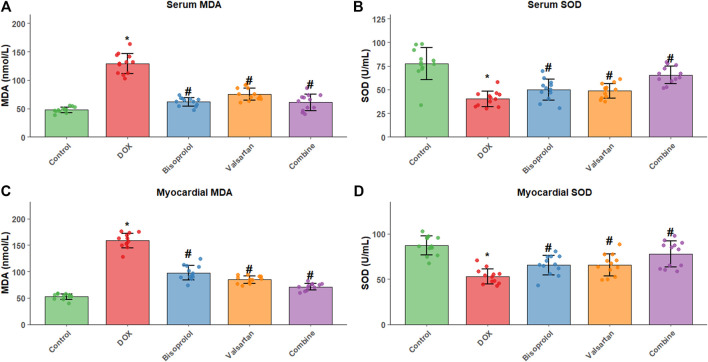
Oxidative stress markers in serum and myocardial tissue. **(A)** Serum MDA, **(B)** serum SOD, **(C)** myocardial MDA, and **(D)** myocardial SOD. Malondialdehyde (MDA) levels and superoxide dismutase (SOD) activity were measured in serum and myocardial tissue from all groups (n = 12 per group). Data are presented as mean ± SD. *P < 0.05 vs. Control group; #P < 0.05 vs. DOX group. The combination group showed the most significant improvement in oxidative stress parameters.

Relative to the Control group, myocardial and serum malondialdehyde (MDA) levels were significantly elevated in the DOX group, whereas superoxide dismutase (SOD) activity was significantly reduced (P < 0.05). Compared with the DOX group, MDA levels were significantly decreased and SOD activity was significantly increased in the Bisoprolol, Valsartan, and combination groups (all P < 0.05). Consistent with other findings, the most substantial improvement in oxidative stress status was observed in the combination group (Figure 5).

### Expression of oxidative stress–related proteins in myocardial tissue

**FIGURE 6:**
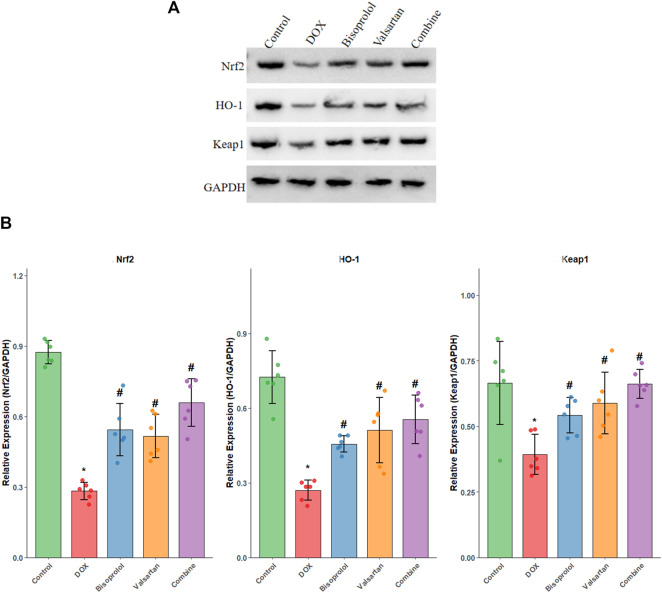
Protein expression of Nrf2, HO-1, and Keap1 in myocardial tissue. **(A)** Representative Western blot bands showing protein expression of Nrf2, HO-1, Keap1, and GAPDH (loading control) in left ventricular tissue from Control, DOX, Bisoprolol, Valsartan, and Combination groups. Each lane represents a sample from each experimental group (n = 6 per group). **(B)** Quantitative analysis of Western blot results showing relative protein expression levels of Nrf2, HO-1, and Keap1 normalized to GAPDH. Individual data points represent the quantified protein expression levels for each experimental animal (n = 6 per group), demonstrating the distribution and variability within each group. Data are presented as mean ± SD. *P < 0.05 vs. Control group; #P < 0.05 vs. DOX group (one-way ANOVA followed by LSD t-test). The combination therapy showed the most significant upregulation of all three antioxidant pathway proteins compared to the DOX group.

Compared with the Control group, protein expression levels of nuclear factor erythroid 2-related factor 2 (Nrf2), haem oxygenase-1 (HO-1), and Kelch-like ECH-associated protein 1 (Keap1) were significantly reduced in the DOX group (P < 0.05). In contrast, expression of Nrf2, HO-1, and Keap1 was significantly upregulated in the Bisoprolol, Valsartan, and combination groups compared with the DOX group (all P < 0.05). The greatest increase in protein expression was again observed in the combination group (Figure 6).

## Discussion

Doxorubicin is an effective chemotherapeutic agent for the treatment of various malignancies; however, its clinical application is significantly limited by severe cardiotoxicity, which adversely affects patients’ quality of life and long-term prognosis [18–20]. In this study, a rat model of doxorubicin-induced cardiotoxicity was established. Following doxorubicin administration, rats exhibited markedly reduced food intake and spontaneous activity, slowed responsiveness, and lethargy. Cardiac dysfunction was evident, accompanied by abnormal myocardial enzyme levels and varying degrees of oxidative stress in myocardial tissue. Histological analysis with H&E staining showed disordered myocardial fibre arrangement and extensive cardiomyocyte necrosis, consistent with previous findings [21], confirming the successful establishment of the cardiotoxicity model. Oxidative stress is a well-recognized mechanism underlying doxorubicin-induced cardiotoxicity. Doxorubicin acts as a highly redox-active substrate; during its metabolism, the quinone moiety undergoes redox cycling, generating excessive reactive oxygen species. These free radicals cause sarcoplasmic reticulum calcium leakage and structural damage to DNA, RNA, proteins, and lipids. Through activation of oxidative stress pathways, doxorubicin disrupts cardiomyocyte integrity, promotes apoptosis and myocardial fibrosis, impairs cardiac function, and ultimately leads to irreversible myocardial injury [22–24].

Several studies have demonstrated that early prophylactic administration of cardioprotective agents can effectively improve cardiac function and prolong survival in patients receiving doxorubicin, particularly with β-adrenergic blockers and angiotensin-converting enzyme inhibitors [25]. Sacubitril/valsartan improves cardiac function through a dual mechanism of neprilysin inhibition and angiotensin receptor blockade, thereby reducing vasoconstriction and attenuating myocardial fibrosis and hypertrophy [26, 27]. Experimental evidence has confirmed that sacubitril/valsartan can alleviate doxorubicin-induced myocardial fibrosis in rats, likely through modulation of oxidative stress pathways [28]. Bisoprolol, a selective β-adrenergic blocker, reduces myocardial oxygen consumption and improves cardiac perfusion by blocking cardiac β-receptors [29, 30]. Previous studies have shown that bisoprolol improves cardiac function and ventricular remodelling in heart failure models by regulating oxidative stress and apoptosis, thereby delaying myocardial fibrosis [31, 32]. However, evidence regarding the combined effects of bisoprolol and sacubitril/valsartan on doxorubicin-induced cardiotoxicity is limited. In the present study, analysis of the combination therapy revealed that, compared with the DOX group, rats in the Bisoprolol, Valsartan, and combination groups exhibited significant improvements in general condition and cardiac function. Histopathological examination further showed attenuation of myocardial injury, with the combination therapy producing the most pronounced protective effect. The observed synergy may be attributed to the dual neurohormonal modulation by sacubitril/valsartan, which inhibits angiotensin II receptors and enhances neprilysin activity to reduce vasoconstriction and promote vasodilation [33]. Meanwhile, bisoprolol, as a highly selective β-blocker, directly suppresses sympathetic activation induced by doxorubicin, thereby reducing myocardial oxygen demand [34]. The combination of these mechanisms likely cooperatively attenuates myocardial fibrosis and improves cardiac function in doxorubicin-treated rats.

Abnormal myocardial enzyme levels and oxidative stress imbalance are closely associated with doxorubicin-induced cardiotoxicity. During its metabolism, doxorubicin generates excessive reactive oxygen species (ROS), disrupting the myocardial oxidative–antioxidative balance. This imbalance compromises the integrity of cardiomyocyte membranes, leading to elevated myocardial enzymes and, ultimately, impaired cardiac function [35]. These observations underscore the pivotal role of oxidative stress in doxorubicin-induced cardiotoxicity.

In the present study, treatment with Bisoprolol, Valsartan, or their combination significantly reduced myocardial CK-MB, cTnI, and BNP levels, as well as ROS fluorescence intensity and malondialdehyde (MDA) content in both myocardial tissue and serum, while superoxide dismutase (SOD) activity was markedly increased. Importantly, the detection of ROS in circulating EV-enriched fractions by flow cytometry provided a complementary “liquid biopsy” assessment of systemic oxidative stress. It should be acknowledged that this method, based on scatter gating, cannot definitively distinguish extracellular vesicles from other submicron particles such as lipoproteins or protein aggregates, and therefore the results should be interpreted as ROS associated with an EV-enriched particle population rather than pure EVs. Nevertheless, the observed correlation between serum particle-associated ROS and intramyocardial oxidative status supports the utility of this method as a complementary indicator of systemic oxidative stress. Among the treatments, the combination therapy produced the most pronounced improvements, indicating that co-administration effectively attenuates myocardial injury. Although single-drug interventions improved oxidative stress and myocardial enzyme profiles, the combined therapy was superior, likely due to its synergistic reduction of ROS generation, inhibition of lipid peroxidation, and enhancement of antioxidant enzyme activity, thereby mitigating oxidative injury to cardiomyocytes and normalizing myocardial enzyme expression. The Nrf2/HO-1 signalling pathway is a key regulator of oxidative stress, and previous studies have shown that its activation can ameliorate doxorubicin-induced cardiotoxicity and delay the progression of myocardial fibrosis in rats [36–37]. Consistently, in this study, Bisoprolol, Valsartan, and their combination upregulated Nrf2, HO-1, and Keap1 protein expression, further demonstrating that the combined therapy mitigates doxorubicin-induced cardiotoxicity through modulation of oxidative stress pathways.

In summary, sacubitril/valsartan combined with bisoprolol attenuates doxorubicin-induced cardiotoxicity in rats via regulation of oxidative stress, thereby improving cardiac function and myocardial enzyme profiles. These findings provide experimental support for potential clinical strategies to prevent doxorubicin-related cardiac injury.

## Data Availability

The original contributions presented in the study are included in the article/supplementary material, further inquiries can be directed to the corresponding authors.
